# Neonatal hyperoxia exposure causes cerebellar lesions and behavioral abnormalities in rats

**DOI:** 10.1038/s41598-025-34530-1

**Published:** 2026-01-12

**Authors:** Sakiko Suzuki, Takahiro Kanzawa, Ryoko Shimode, Yukina Takamoto, Kazuto Ueda, Ryosuke Miura, Toshihiko Suzuki, Naoki Tajiri, Hideki Hida, Yoshiyuki Takahashi, Masahiro Hayakawa, Yoshiaki Sato

**Affiliations:** 1 Division of Neonatology, Center for Maternal-Neonatal Care, Nagoya University Hospital, 65 Tsurumai-cho, Showa-ku, Nagoya, Aichi 466-8560 Japan; 2 Department of Pediatrics, Nagoya University Graduate School of Medicine, Nagoya, Japan; 3 Department of Neurophysiology & Brain Science, Nagoya City University Graduate School of Medical Sciences & Medical School, Nagoya, Japan

**Keywords:** Neurologic manifestations, Problem behavior, Cerebellum, Infant, Premature, Cell movement, Purkinje cells, Neuroscience, Neurology

## Abstract

In humans, fetal cerebellar development peaks during the final stages of pregnancy. Preterm infants experience hyperoxia (excessive oxygen) outside the uterus. Hyperoxia exposure causes neurological deficits in preterm infants. However, detailed mechanisms underlying hyperoxia-induced neurological deficits remain unclear. Previous studies on neurological deficits have focused on cerebral lesions. However, recently, cerebellar lesions have been observed on brain magnetic resonance imaging in preterm infants. We herein aimed to investigate behavioral and cerebellar tissue–level changes in Sprague–Dawley rat neonates exposed to 83% hyperoxia from within 24 h of birth to 14 days of age. In rats, cerebellar development peaks in the first postnatal week. We elucidated the mechanism by which hyperoxia exposure causes neurological deficits in these rats. We found that prolonged hyperoxia exposure, starting within 24 h of birth, induces behavioral impairments (such as motor, cognitive and memory, and social interaction deficits) in rats. At the tissue level, delayed granule cell migration and abnormal Purkinje cell dendritic development combined with impaired myelination were observed in the acute and chronic hyperoxia exposure phases, respectively. Thus, hyperoxia exposure may cause abnormalities in cerebellar morphology and function, resulting in neurological deficits in preterm infants.

**Supplementary Information:**

The online version contains supplementary material available at 10.1038/s41598-025-34530-1.

## Introduction

Although recent advances in neonatal care have increased the survival rate of infants with extremely-low birthweight (weighing < 1000 g), the incidence of neurological disorders has remained high^1,2^. Moreover, the high incidence of developmental disorders, such as autism spectrum disorder (ASD), attention deficit hyperactivity disorder, and learning disabilities, as well as cerebral palsy and intellectual disability is concerning^3–7^.

Traditionally, studies on neurological disorders have primarily focused on cerebral lesions. However, recently, brain magnetic resonance imaging revealed that a high percentage of preterm infants has cerebellar lesions^8–11^. The cerebellum is involved in motor coordination as well as learning, perception, and other higher brain functions; moreover, it is implicated in developmental disorders, such as ASD^12–14^. Normally, human cerebellar development peaks in the third trimester of pregnancy, when cells in the external granular layer proliferate, migrate to the internal granular layer, and form the gyrus; in addition, Purkinje cells mature during this period^15^. Therefore, it is highly likely that the abnormal brain function observed in preterm infants is caused by impaired cerebellar development that occurs during this period.

Perinatal tissue oxygen concentrations increase more rapidly in preterm infants than in the normal developmental environment in utero, even under room air conditions^16^. In addition, many preterm infants require supplemental oxygen during resuscitation. Moreover, many preterm infants, particularly those with bronchopulmonary dysplasia (BPD), are chronically exposed to high concentrations of oxygen (hyperoxia). Because fetal development occurs under hypoxic conditions in utero^17^, hyperoxia may cause neurological damage in preterm infants^16^. However, the mechanisms underlying hyperoxia-induced neurological deficits remain unclear.

We herein aimed to determine the neurological deficits (behavioral and histological) related to hyperoxia exposure. Among the various neurological deficits observed in preterm infants, we focused on cerebellar impairment resulting from hyperoxia exposure. In this study, using our previously developed BPD rat model (which shows lung injury from hyperoxia exposure)^18^, we evaluated the neurological deficits caused by hyperoxia exposure in neonatal rat pups.

## Results

### Evaluation of behavioral tests

#### Neonatal hyperoxia exposure caused impaired primitive reflexes in negative geotaxis test

To assess the maturity of vestibular receptors, central sensory function, and motor function, we conducted a negative geotaxis test for 8 consecutive days, i.e., from postnatal day (P) 8 to P15^19^. The time taken to complete the 180° upward turn was scored on a scale of 0–5, with a lower score indicating a longer time to turn.

**Fig. 1:**
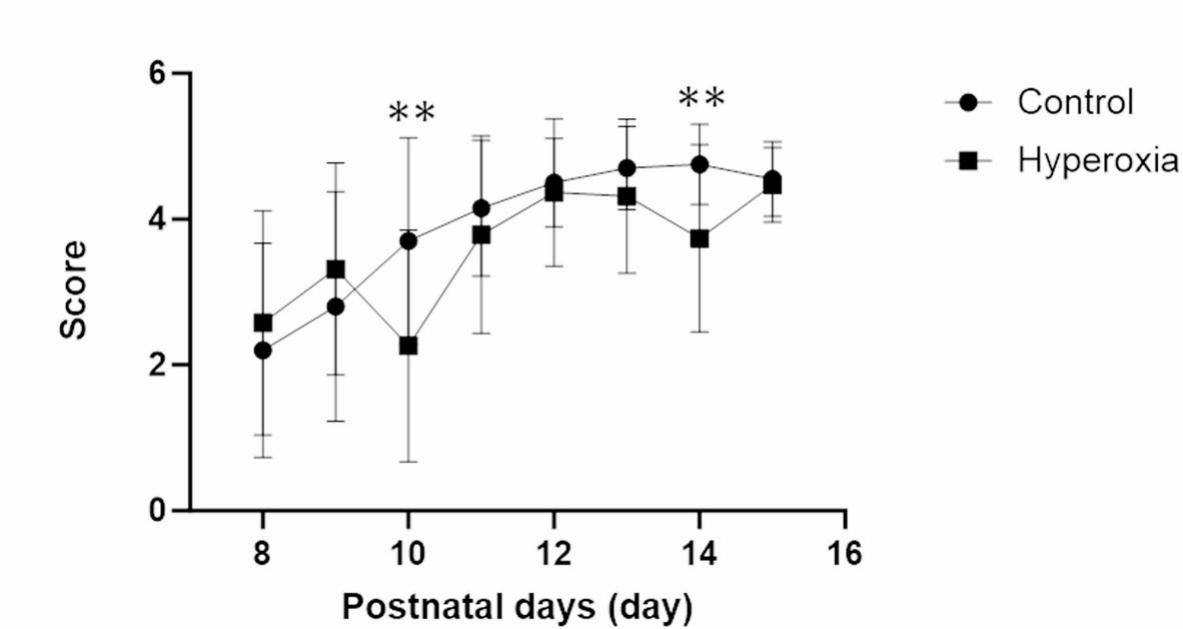
Neonatal hyperoxia exposure affects negative geotaxis behavior. At postnatal day (P) 10 and P14, the score (indicating the time required to make the 180° turn) was significantly lower (i.e., the rats took longer to make the turn) in the hyperoxia group than in the control group (scored on a scale of 0–5 based on the time required to make the 180° turn; score 5, 0–15 s; score 4, 15–30 s; score 3, 30–45 s; score 2, 45–60 s; score 1, > 60 s or drop; score 0, no response; control, *n* = 20; hyperoxia, *n* = 19). Data represent mean ± standard deviation; **p < 0.01.

Repeated measures analysis of variance (ANOVA) revealed a significant Time × Group interaction (F = 4.694, *p* < 0.01), indicating differences in negative geotaxis scores between the control and hyperoxia groups. Post hoc analysis using Bonferroni-corrected t-tests confirmed that the hyperoxia group had significantly lower scores than the control group at P10 and P14 (adjusted *p* = 0.01 and < 0.01, respectively) (Fig. 1). In particular, at P10 and P14, the mean scores were 3.7 ± 1.4 (95% confidence interval [CI]: 3.0–4.4) versus 2.3 ± 1.6 (95% CI 1.5–3.0) (control vs. hyperoxia, *p* < 0.01, uncorrected) and 4.8 ± 0.6 (95% CI 4.5–5.0) versus 3.7 ± 1.3 (95% CI 3.1–4.4) (*p* < 0.01, uncorrected), respectively.

#### Neonatal hyperoxia exposure did not affect general behavior in the open field test

**Fig. 2:**
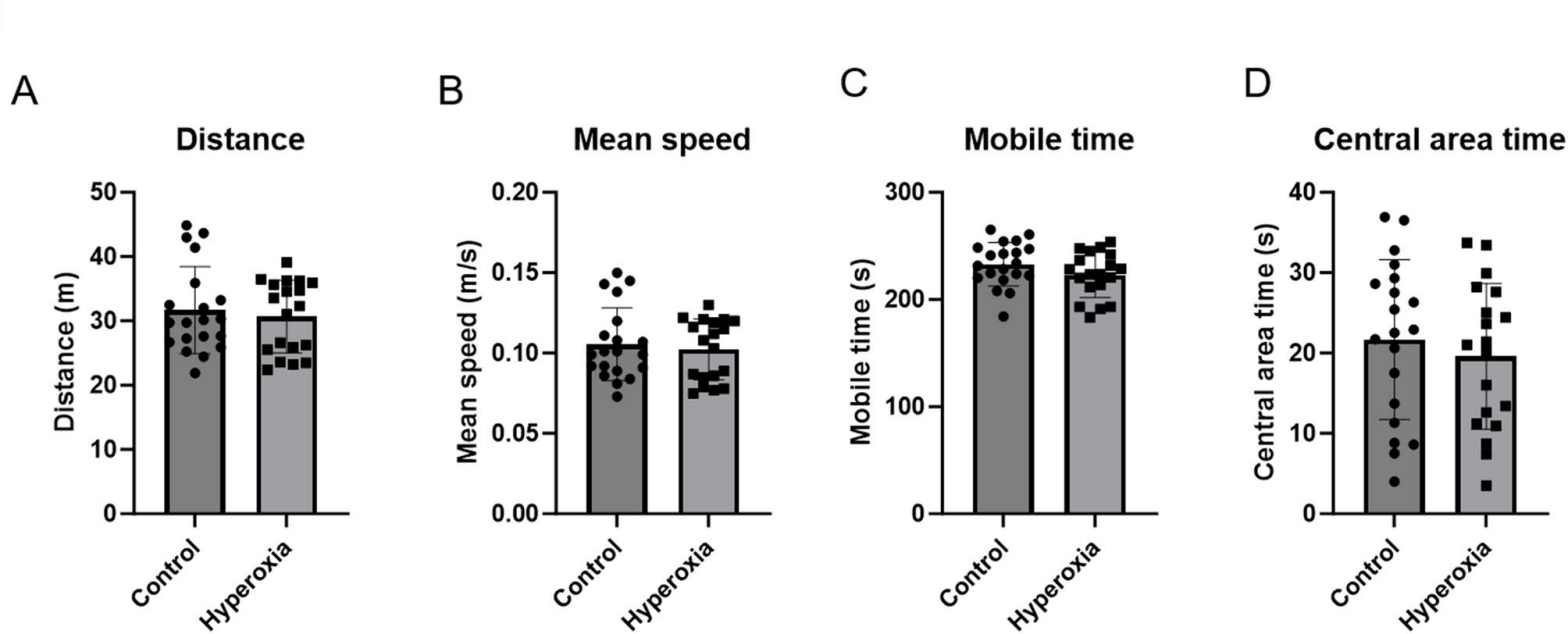
General behavior in the open field test was not affected by neonatal hyperoxia exposure. (**A**) Distance; (**B**) Mean speed; (**C**) Mobile time; and (**D**) Central area time. The parameters were not significantly different between the control group and hyperoxia group (control, *n* = 20; hyperoxia, *n* = 19).

To assess general behaviors such as hyperactivity and behavioral abnormalities, we conducted open field tests using 5-week-old rats. No significant differences were observed between the two groups in any of the tested parameters: distance (control vs. hyperoxia, 31.7 ± 6.8 m [95% CI 28.5–34.8] vs. 30.7 ± 5.7 m [95% CI 28.0–33.4]), mean speed (control vs. hyperoxia, 0.11 ± 0.02 m/s [95% CI 0.09–0.12] vs. 0.10 ± 0.02 m/s [95% CI 0.09–0.11]), mobile time (control vs. hyperoxia, 232.9 ± 20.3 s [95% CI 223.4–242.4] vs. 222.9 ± 21.1 s [95% CI 212.7–233.1]), and central area time (control vs. hyperoxia, 21.7 ± 9.9 s [95% CI 17.0–26.3] vs. 19.6 ± 9.1 s [95% CI 15.2–24.0]) (Fig. 2A–D).

#### Neonatal hyperoxia exposure caused motor deficits affecting performance in the horizontal ladder walking test

**Fig. 3:**
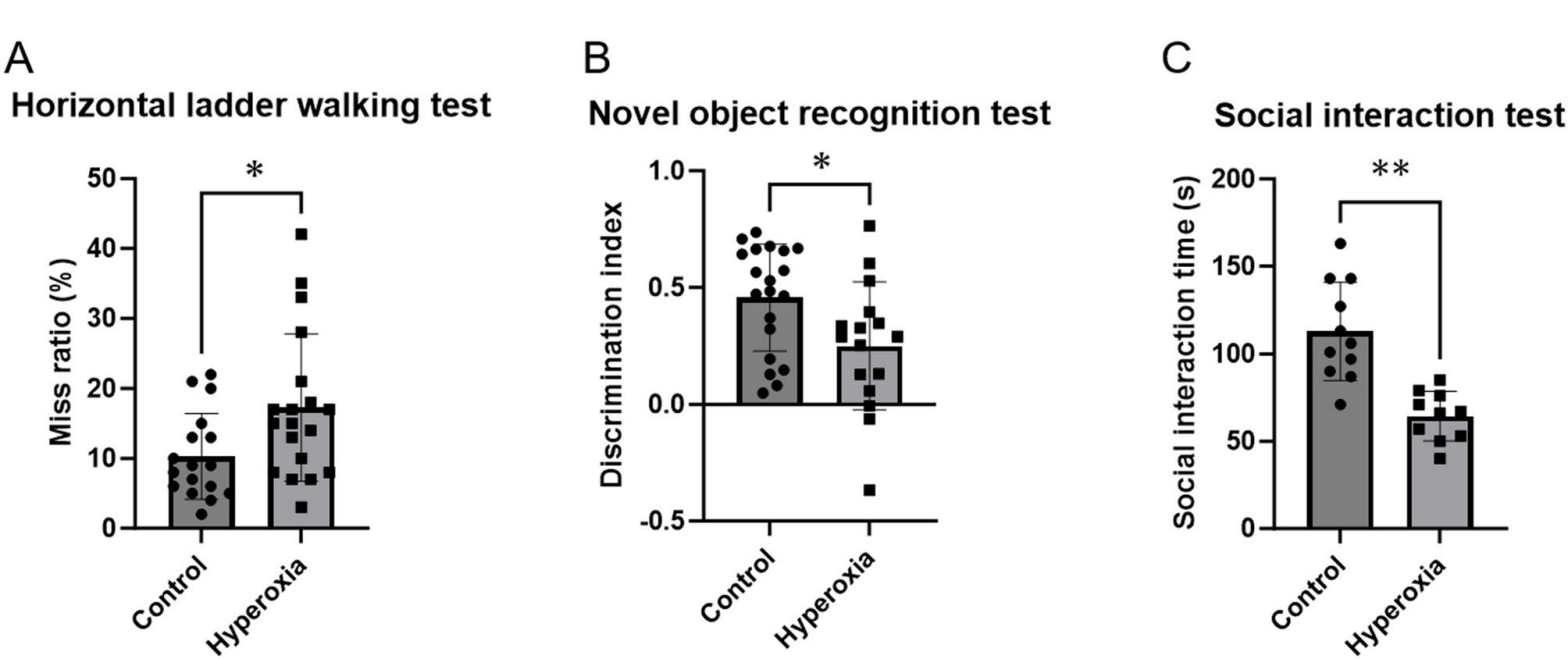
Neonatal hyperoxia exposure affected motor coordination, cognitive and memory abilities, and social interaction. (**A**) Horizontal ladder walking test: The proportion of missed steps was significantly higher in the hyperoxia group than in the control group (control, *n* = 17; hyperoxia, *n* = 19). (**B**) Novel object recognition test: Discrimination index was significantly lower in the hyperoxia group than in the control group (control, *n* = 20; hyperoxia, *n* = 16). (**C**) Social interaction test: Social interaction duration was significantly shorter in the hyperoxia group than in the control group (control, *n* = 11; hyperoxia, *n* = 13). Data represent mean ± standard deviation; *p < 0.05 and **p < 0.01.

To assess motor deficits, we conducted a horizontal ladder walking test using 7-week-old rats. The hyperoxia group exhibited a significantly higher proportion of missed steps than the control group (Fig. 3A; control vs. hyperoxia, 10.3% ± 6.1% [95% CI 7.1–13.5%] vs. 17.3% ± 10.5% [95% CI 12.2–22.3%]; *p* < 0.05).

#### Neonatal hyperoxia exposure affected cognitive and memory abilities, as evaluated using the novel object recognition test

To assess cognitive and memory abilities, we conducted a novel object recognition test using 6-week-old rats. The hyperoxia group exhibited a significantly lower discrimination index than the control group (Fig. 3B; control vs. hyperoxia, 0.46 ± 0.23 [95% CI 0.35–0.56] vs. 0.25 ± 0.27 [95% CI 0.11–0.40], *p* < 0.05).

#### Neonatal hyperoxia exposure affected social skills, as evaluated using the social interaction test

To assess social skills, we conducted a social interaction test using 7-week-old rats. The hyperoxia group showed significantly shorter social interaction duration than the control group (Fig. 3C; control vs. hyperoxia, 112.8 ± 28.1 s [95% CI 93.9–131.7] vs. 64.4 ± 14.2 s [95% CI 54.3–74.6], *p* < 0.01).

### Evaluation of cerebellar histological changes during acute phase

**Fig. 4:**
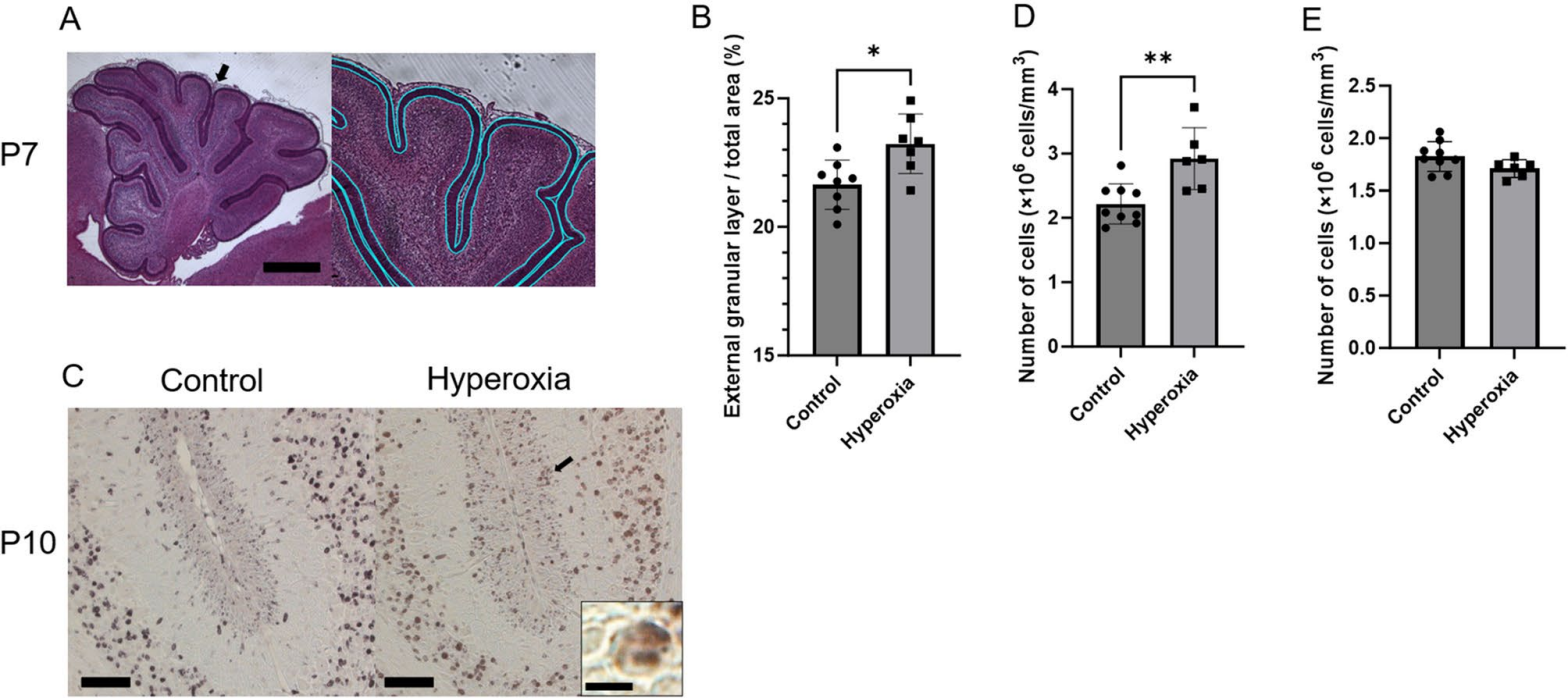
Neonatal hyperoxia exposure affected migration of granular cells in the external granular layer. (**A**) Representative photomicrographs of the external granular layer in the P7 cerebellar tissue section (Bar = 500 μm). The right panel shows a higher-magnification view. The black arrow indicates the magnified area. The fluorescent blue line surrounds the external granular layer. (**B**) Ratio of the external granular layer volume to the total cerebellar hemisphere volume at P7 was significantly higher in the hyperoxia group than in the control group (control, *n* = 8; hyperoxia, *n* = 7). (**C**) Representative photomicrographs of the P10 cerebellar tissue section showing BrdU-positive cells (Bar = 50 μm; Insets show higher-magnification views [bar = 5 μm]; Black arrow indicates the area magnified). (**D**) The number of BrdU-positive cells in the inner margin of the external granular layer at P10 was significantly higher in the hyperoxia group than in the control group (control, *n* = 9; hyperoxia, *n* = 6). (**E**) The number of BrdU-positive cells in the internal granular layer at P10 was not significantly different between the control and hyperoxia groups (control, *n* = 9; hyperoxia, *n* = 6). Data represent mean ± standard deviation; *p < 0.05 and **p < 0.01.

Figure 4A shows representative photomicrographs of the external granular layer in the P7 cerebellar tissue sections. At P7, the ratio of the external granular layer volume to the total cerebellar hemisphere volume was significantly higher in the hyperoxia group than in the control group (Fig. 4B; control vs. hyperoxia, 21.6% ± 0.96% [95% CI 20.8–22.4%] vs. 23.2% ± 1.15% [95% CI 22.2–24.3%]; *p* < 0.05). Representative photomicrographs of the P10 cerebellar tissue sections showed BrdU-positive cells in the inner margin of the external granular layer (Fig. 4C). The hyperoxia group had significantly more BrdU-positive cells in the inner margin of the external granular layer than the control group (Fig. 4D; control vs. hyperoxia, 2.2 ± 0.31 [95% CI 2.0–2.5] ×10^6^ cells/mm^3^ vs. 2.9 ± 0.48 [95% CI 2.4–3.4] ×10^6^ cells/mm^3^, *p* < 0.01). Meanwhile, the P10 cerebellar tissue sections of the control group showed an insignificant increase in the number of BrdU-positive cells in the internal granular layer compared with those in the hyperoxia group (Fig. 4E; control vs. hyperoxia, 1.8 ± 0.14 [95% CI 1.7–1.9] vs. 1.7 ± 0.08 [95% CI 1.6–1.8] ×10^6^ cells/mm^3^, *p* = 0.097). These findings indicated that granular cell migration was delayed in the hyperoxia group.

### Evaluation of cerebellar histological changes during chronic phase

**Fig. 5:**
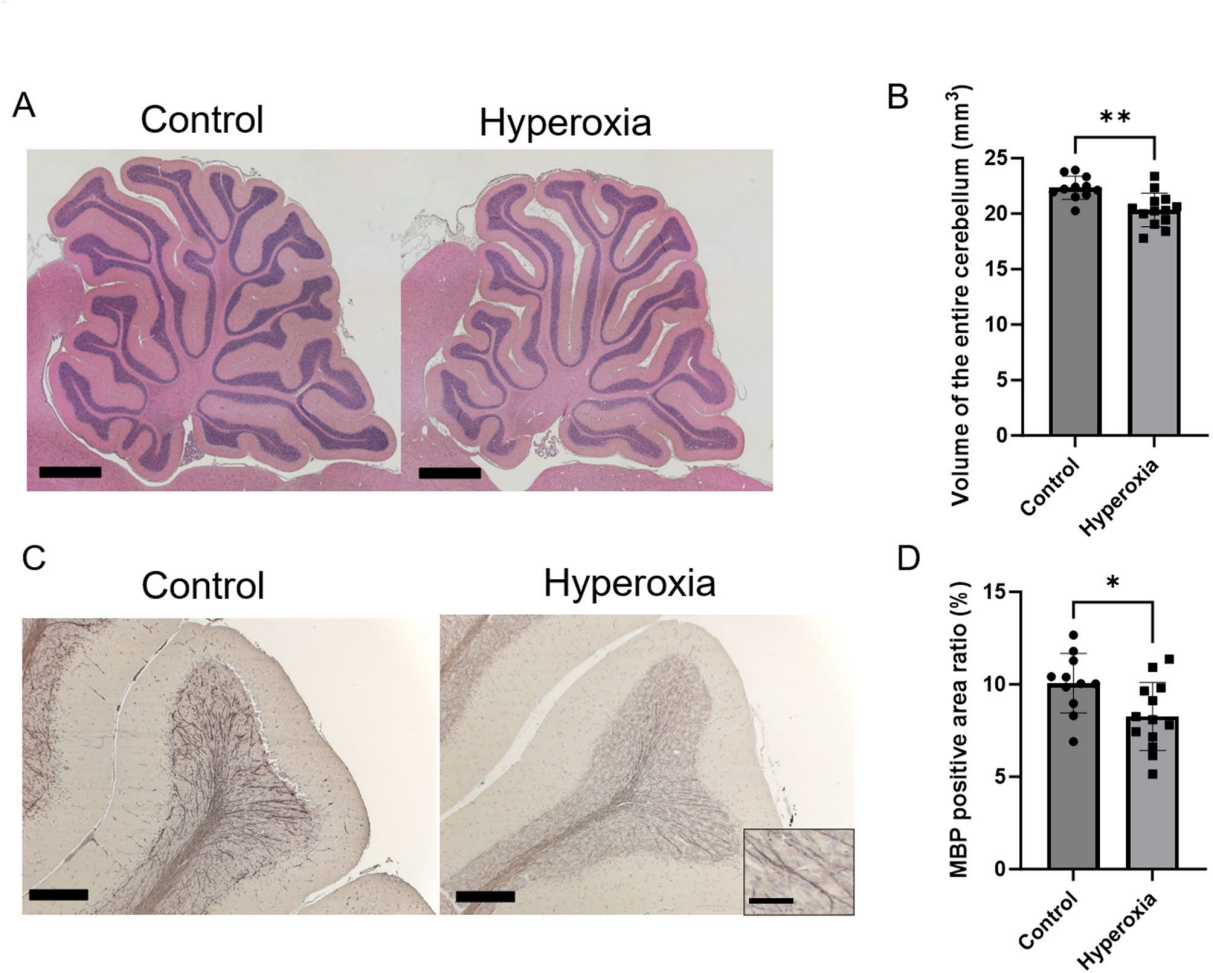
Neonatal hyperoxia exposure reduced cerebellar volume and affected myelin sheath growth. (**A**) Representative photomicrographs of the section stained with hematoxylin and eosin (HE) (Bar = 1000 μm). (**B**) Total cerebellar volume was significantly lower in the hyperoxia group than in the control group (control, *n* = 11; hyperoxia, *n* = 10). (**C**) Representative photomicrographs of the section stained with myelin basic proteins (MBPs) (Bar = 200 μm; Insets show higher-magnification views [Bar = 50 μm]). (**D**) The ratio of areas positive for MBPs in the hyperoxia group was significantly lower than that in the control group (control, *n* = 11; hyperoxia, *n* = 13). Data represent mean ± standard deviation; *p < 0.05 and **p < 0.01.

**Fig. 6:**
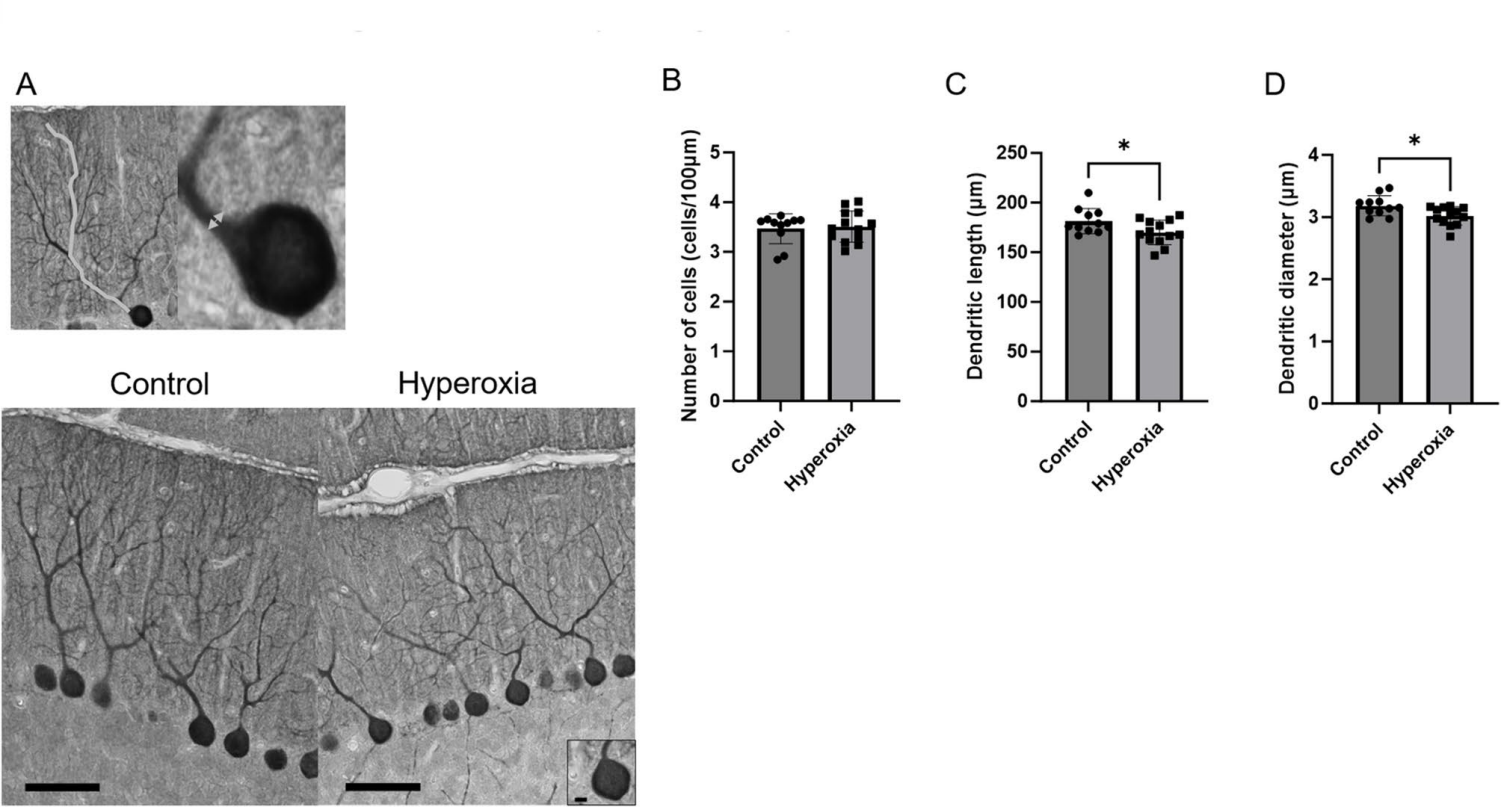
Neonatal hyperoxia exposure reduced Purkinje cell dendritic length and diameter. (**A**) Representative photomicrographs of Calbindin-stained Purkinje cells (Bar = 50 μm; Insets show higher-magnification views [Bar = 5 μm]). The gray line and arrow show the length and diameter of the dendrites, respectively. (**B**) The number of Purkinje cells per 100 μm was not significantly different between the two groups (control, *n* = 11; hyperoxia, *n* = 13). (**C**) The dendritic length of Purkinje cells in the hyperoxia group was significantly shorter than that in the control group (control, *n* = 11; hyperoxia, *n* = 13). (**D**) The dendritic diameter of Purkinje cells in the hyperoxia group was significantly shorter than that in the control group (control, *n* = 11; hyperoxia, *n* = 13). Data represent mean ± standard deviation; *p < 0.05.

Figure 5A shows representative photomicrographs of the total cerebellar volume. The hyperoxia group showed significantly lower cerebellar volume than the control group (Fig. 5B; control vs. hyperoxia, 22.3 ± 2.0 mm^3^ [95% CI 21.6–23.0] vs. 20.3 ± 1.5 mm^3^ [95% CI 19.4–21.3], *p* < 0.01). Representative photomicrographs of areas of cerebellar tissue sections stained positive for myelin basic proteins (MBPs) are shown in Fig. 5C. The ratio of area positive for MBPs to the entire cerebellar tissue section area was significantly lower in the hyperoxia group than in the control group (Fig. 5D; control vs. hyperoxia, 10.1% ± 1.6% [95% CI 9.0–11.1%] vs. 8.3% ± 1.8% [95% CI 7.2–9.4%], *p* < 0.05). In addition, representative photomicrographs of Calbindin-stained Purkinje cells are presented in Fig. 6A. The number of Purkinje cells per 100 μm was not significantly different between the two groups (control vs. hyperoxia, 3.5 ± 0.3 cells/100 µm [95% CI 3.3–3.7] vs. 3.5 ± 0.3 cells/100 µm [95% CI 3.3–3.7], *p* = 0.7527). However, the Purkinje cell dendritic length (control vs. hyperoxia, 181.4 ± 12.6 μm [95% CI 172.9–189.9] vs. 169.9 ± 12.4 μm [95% CI 162.4–177.4], *p* < 0.05) and diameter (control vs. hyperoxia, 3.2 ± 0.2 μm [95% CI 3.1–3.3] vs. 3.0 ± 0.1 μm [95% CI 2.9–3.1], *p* < 0.05) were significantly reduced in the hyperoxia group compared with those in the control group (Fig. 6B–D).

## Discussion

In the present study, we identified behavioral abnormalities and cerebellar lesions in a neonatal hyperoxia exposure rat model. In terms of behavior, we observed the following abnormalities in neonatal hyperoxia exposure model rats with cerebellar dysfunction: (1) significant delay in negative geotaxis, which is a primitive reflex, in the acute phase and (2) motor, cognitive and memory, and social deficits in the chronic phase. Furthermore, histologically, we observed the following cerebellar lesions in neonatal hyperoxia exposure model rats with cerebellar dysfunction: (1) delayed granule cell migration in the acute phase and (2) reduced myelination and abnormal dendritic development of Purkinje cells as well as reduced cerebellar volume in the chronic phase.

A previous study on hyperoxia-induced neurological deficits focused on hyperoxia occurring in 1-week-old rats, with shorter study durations lasting from a few hours to a few days^17^. However, in the present study, we exposed neonates to hyperoxia within 24 h of birth and maintained the exposure over longer durations compared with those in earlier studies^17^. Although neurological deficits have been reported in previous studies^20–24^, our neonatal hyperoxia exposure protocol is more suitable for examining the pathophysiology of preterm infants exposed to hyperoxia immediately after birth (and continuously) in an environment different from that in utero. In addition, many preterm infants require oxygen supplementation during resuscitation and are often chronically exposed to hyperoxia. In particular, preterm infants with BPD are more likely to be exposed to hyperoxia for longer periods. Similar behavioral abnormalities associated with cerebellar dysfunction were observed in our neonatal hyperoxia-exposed rat model. Because cerebellum is crucial for many neurological functions such as motor coordination, learning, and perception as well as other higher brain functions, impaired cerebellar development may cause several neurological deficits. Hyperoxia exposure may affect cerebellar development in preterm infants, because they are born prematurely during a critical period of cerebellar development and placed in an external environment, with conditions different from the uterus. In rats, cerebellar development peaks in the first postnatal week^15,23,25^. Thus, exposing rat neonates, within 24 h of their birth, to hyperoxia can recapitulate the cerebellar development occurring in preterm infants fairly well. Therefore, our neonatal hyperoxia exposure rat model is suitable for studying hyperoxia-induced impairment of cerebellar development in preterm infants.

Consistent with previous studies^24,26,27^, our behavioral evaluations demonstrated that rats neonatally exposed to hyperoxia exhibited impairments in motor, cognitive, and memory functions as well as social deficits. However, unlike the findings of the other behavioral assessments in this study, no significant difference was observed between the control and hyperoxia groups in the open field test, which evaluates general behavior in rats. This could be because the 5-week-old adolescent rats used in the tests are typically more hyperactive than adult rats, making it difficult to detect subtle variations in general behavior, such as hyperactivity (as is the case in the model studied here). Notably, previous studies examining hyperoxia-induced behavioral abnormalities found no differences in performance in the open field test^24,26^, implying that hyperoxia-induced brain damage may not be sufficient to cause general behavioral changes.

Furthermore, our histological evaluations revealed that rats neonatally exposed to hyperoxia had cerebellar lesions. The neonatal rat pup cerebellum has a layered structure comprising the external granular layer, molecular layer, Purkinje cell layer, internal granular layer, and white matter; the external granular layer is absent in adult rats^28^. Active cell division occurs in the external granular layer. Around P7, proliferating cells gradually migrate to the internal granular layer, eventually forming the adult granular cell layer. In rats, the external granule layer mostly develops between P4 and P10^28–30^. Therefore, we first evaluated the ratio of the area of the external granular layer to the total area of the cerebellar hemispheres in the cerebellar tissue collected at P7; this ratio was high in the neonatal hyperoxia exposure model rats. Second, we evaluated the proliferation and migration of granular cells in the cerebellar tissues of P10 rat pups intraperitoneally injected with BrdU at P6. The rats exposed neonatally to hyperoxia had more BrdU-positive cells in the inner margin of the external granular layer and tended to have fewer BrdU-positive cells in the internal granular layer. BrdU stains proliferating cells by entering their DNA during the S phase of the cell cycle. Actively proliferating cells remain in the uppermost part of the external granular layer for 20–48 h after their final division before migrating radially across the molecular layer, guided by the processes of Bergmann glial cells^28,31^. Our results indicate that hyperoxia induces increased cell proliferation in the external granular layer and/or delays cell migration from the external granular layer to the internal granular layer. Both mechanisms can lead to a larger external granular layer area, as observed in this study. Although the detailed mechanism of these hyperoxia-induced changes is unclear, during cerebellar development, granule cells proliferating in the external granular layer migrate to the internal granular layer while extending parallel fibers to form synapses with the dendritic processes of Purkinje cells. Thus, delayed granule cell migration may cause abnormal synaptogenesis along the Purkinje cell dendrites, which in turn may cause cerebellar dysfunction.

Histological evaluation of the cerebellum of adult rats neonatally exposed to hyperoxia revealed decreased cerebellar volume, decreased myelination, and abnormal development of Purkinje cell dendrites. The adult rat cerebellum lacks an external granular layer and has a layered structure comprising the molecular layer, Purkinje cell layer, granule cell layer, and white matter layer. Purkinje cells are the main neurons in the cerebellum, and they are the only output cells that transmit information processed in the cerebellum to the higher brain structures. Dendrites of Purkinje cells develop through repeated branching while synapsing with parallel fibers (the axons of internal granule cells)^32,33^. Thus, the cerebellar volume reduction observed in hyperoxia-exposed rats can be attributed to its effects on either of the cerebellar layers. We propose that hyperoxia affects the granule cell layer, as evidenced by the delayed granule cell migration (as observed in this study), which might cause the final internal granular layer to have a lower number of granule cells, consequently affecting the cerebellar volume. Regarding the effects of hyperoxia on Purkinje cells and the molecular cell layers, hyperoxia did not affect the number of Purkinje cells in the adult cerebellum of rats neonatally exposed to hyperoxia. However, the length and diameter of the Purkinje cell dendrites were reduced, indicating that hyperoxia causes Purkinje cell dendrites in the molecular cell layer to develop abnormally, possibly affecting the cerebellar volume. Purkinje cell development may be affected by hyperoxia directly or by delaying granule cell migration. Migrating granule cells play a crucial role in the maturation of Purkinje cells^23^. Previous studies have indicated that hyperoxia reduces the diameter of myelinated axons and the expression of myelin proteins; furthermore, it causes abnormal myelin structure and alters the phosphorylation of neurofilaments^34,35^. Our results showed that hyperoxia exposure shrinks the areas of the regions expressing MBPs in the cerebellum.

The excitatory–inhibitory balance plays a crucial role in cerebellar development and function^36^. Purkinje cells, the only output cells of the cerebellum, regulate their activity by integrating excitatory and inhibitory input from different neural pathways, including excitatory input from parallel fibers of granule cells and climbing fibers from neurons of the inferior olive nucleus as well as inhibitory input from molecular layer interneurons^36,37^. Purkinje cell dendrites develop through repeated branching and synapse with parallel fibers^32,33^. In the early postnatal period, most Purkinje cells receive inputs from multiple climbing fibers; however, Purkinje cells in the adult cerebellum receive strong synaptic inputs from a single climbing fiber^37^. Thus, synaptogenesis changes with maturation, and abnormal synaptic morphology is strongly associated with many neurological disorders^32,38,39^. Although the present study did not address synapse formation between Purkinje cell dendrites and climbing fibers or GABAergic interneurons in the molecular layer, our findings indicated that hyperoxia exposure may cause abnormalities in the synapse formation between Purkinje cell dendrites and parallel fibers of granule cells. A previous study reported that hyperoxia exposure altered synapse formation in Purkinje celldendrites^23^. Therefore, hyperoxia exposure may have disrupted the balance of excitatory and inhibitory inputs to Purkinje cells, potentially contributing to behavioral abnormalities.

Furthermore, neuronal cell membranes contain Cl^−^ transporters, such as KCC2, which transports Cl^−^ from intracellular to extracellular space, and NKCC1, which transports Cl^−^ from extracellular to intracellular space to maintain [Cl^−^]_i_ homeostasis^40^. In early developing neurons, KCC2 is underdeveloped and [Cl^−^]_i_ is high because of the action of NKCC1, causing GABA to act as an excitatory rather than inhibitory neurotransmitter. However, as development progresses, KCC2 expression increases, whereas NKCC1 expression decreases, resulting in decreased [Cl^−^]_i_ and a shift in GABAergic signaling toward an inhibitory function^41^. Disruptions in Cl^−^ homeostasis during development are associated with the etiology of various psychiatric disorders^42^. Although the current study did not examine GABAergic Purkinje cell maturation, it may be affected in preterm infants born during a critical period of rapid changes in Cl^−^ transporter expression and GABAergic activity and who develop in a hyperoxic environment different from the normal developmental conditions. Further studies are needed to determine the effects of hyperoxia exposure on the maturation of GABAergic Purkinje cells.

Thus, neonatal exposure to hyperoxia may chronically affect cerebellar morphology by modifying the developmental course of the cerebellum, and the consequent impaired cerebellar morphology may contribute to cerebellar dysfunction, leading to behavioral abnormalities. In addition, we used a correlation analysis to investigate the association between morphological parameters and behavioral outcomes. The results are presented in Supplemental Figures (S1–S3). We found a significant correlation between the miss ratio of the horizontal ladder walking test and the percentage of myelin basic protein (MBP)-positive areas. In addition, we found a significant correlation among the social interaction duration of the social interaction test, the volume of the entire cerebellum, and the percentage of MBP-positive areas. These findings indicated that the histological changes observed in our study were related to behavioral abnormalities.

In this study, we found that rats neonatally exposed to hyperoxia exhibited behavioral impairment, particularly impaired social interactions. Cerebellar dysfunction and ASD are related^39,43^. Furthermore, animal models with abnormalities in Purkinje cell dendritic development and synaptogenesis between dendrites and climbing/parallel fibers exhibited impaired motor function and social communication^32^. Furthermore, animal models with impaired granule cell proliferation exhibit abnormalities in cerebellar formation and motor deficits^44^. In this study, we showed that delayed granule cell migration in the acute phase reduced myelination, whereas abnormal development of Purkinje cell dendrites in the chronic phase may impair social interactions, which is a hallmark characteristic of ASD.

The hippocampus plays a key role in cognitive and memory functions, and impairment of the hippocampus cannot be ignored for its effects on cognitive and memory functions. A previous study reported that cerebellar lesions may affect hippocampal function^45^. It has become clear that the cerebellum possesses extensive neural connections not only with the motor and sensory areas of the cerebrum but also with various brain regions responsible for cognitive functions, such as the entire prefrontal cortex, parietal association areas, and limbic system^46^. This suggests that the cerebellum regulates cognition and memory using the same mechanisms it employs to modulate motor control^47^. However, hippocampal lesions were not directly assessed in this study, therefore it is unclear whether the cognitive and memory impairment observed in this study was due to the effects of cerebellar lesions on hippocampal function. Further studies targeting both hippocampal and cerebellar lesions are needed to clarify the relationship between cerebellar lesions and hippocampal function.

The model rats used in this study were BPD model rats that showed lung injury due to high oxygen exposure. Previous studies have showed an association between BPD and neurological prognosis^48,49^. However, the detailed mechanism remains unclear. Hyperoxia exposure associated with BPD and its effects are considered the primary cause affecting neurological developmental outcomes^50^. In addition, high oxygen levels can directly induce oxidative stress and injury in the developing brain, potentially contributing to neurodevelopmental deficits^50^. Meanwhile, the possibility of a complex interorgan relationship, termed the “lung–brain axis,” has also been proposed, implying that lung impairment may influence the brain^51,52^. Further studies are needed to elucidate the detailed mechanisms underlying the association between BPD and neurological developmental outcomes.

This study had limitations. First, we found that hyperoxia causes cerebellar lesions. However, we did not elucidate the detailed molecular mechanisms by which hyperoxia causes these cerebellar lesions. In future, the hypothesized effects of hyperoxia-induced reactive oxygen species and free radicals on cerebellar development should be investigated in detail. Second, sex-based differences in hyperoxia-induced cerebellar damage have not been addressed. To avoid the effects of the female estrous cycle, we conducted behavioral tests only in male rats, whereas histological evaluation of the acute phase was performed only in female rats, which were not used for behavioral tests. Meanwhile, histological evaluation of the chronic phase was limited to male rats because it was performed after behavioral tests. Because sex-based differences in neurological damage (not related to hyperoxia) have been reported^53,54^, additional studies examining sex-based differences in hyperoxia-induced cerebellar damage are required. Third, although we focused on histologically evaluating cerebellar lesions, which have received little attention, the behavioral changes observed in this study cannot be solely attributed to cerebellar lesions. Future studies should examine both cerebral and cerebellar lesions to gain a more precise understanding of the causes of hyperoxia-induced behavioral changes.

## Conclusion

In this study, we revealed that neonatal exposure to hyperoxia in rats causes behavioral abnormalities—including motor, cognitive and memory, and social interaction deficits—and cerebellar lesions—caused by delayed granule cell migration in the acute phase and reduced myelination and abnormal dendritic development of Purkinje cells in the chronic phase. Cerebellar development in the rat neonatal period corresponds to that in preterm infants. Perinatally, preterm infants are exposed to an environment very distinct from the in utero one—particularly in terms of oxygen concentration. Moreover, many preterm infants who receive oxygen supplementation, particularly those with BPD, are chronically exposed to hyperoxia. Our results indicate that hyperoxia in preterm infants may cause cerebellar dysfunction, resulting in neurological deficits, including social communication deficits.

## Methods

### Ethical approval

The animal experiment protocols followed in this study were approved by the Institutional Review Board of Nagoya University, Japan (permit numbers: 20,032, M210107-006, and M220141-002), and were conducted in accordance with the Regulations on Animal Experiments in Nagoya University. This study is reported in compliance with the ARRIVE guidelines (Animal Research: Reporting in Vivo Experiments).

### Animals

Pregnant Sprague–Dawley rats were obtained from Japan SLC Inc. Rats were housed in an environment with a 12-h/12-h light/dark cycle with ad libitum access to water and food. Behavioral evaluations were performed using only male rats to avoid the influence of the estrous cycle. For decreasing animal numbers, only female rats that were not used for behavioral evaluations were used for histological evaluations in the acute phase. Only male rats that were used for behavioral evaluations were used for histological evaluations in the chronic phase. Our previous studies have indicated that the number of samples required for statistical analysis is higher for behavioral evaluation than that for histological evaluation. Therefore, we estimated the sample size required for behavioral evaluation. We determined that the minimum sample size required for the horizontal ladder walking test is 16 (in each group), based on the preliminary experiments conducted to achieve 80% testing power with an error rate of 5%, assuming 10% difference and 10% standard deviation.

### Modeling

**Fig. 7:**
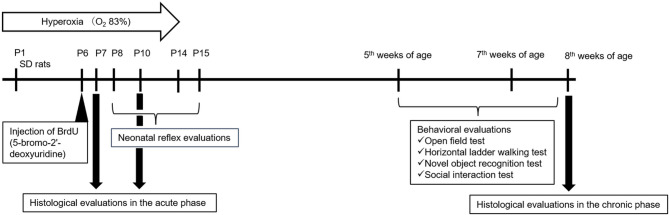
Experimental timeline. Schematic of the experimental protocol. P, postnatal day; SD, Sprague–Dawley.

The rat neonatal hyperoxia exposure model (a BPD model, originally targeting lung injury) was created as previously described^18^. In brief, for hyperoxia exposure, neonatal pups were housed within 24 h of birth in rat cages placed in an 83% hyperconcentrated oxygen chamber until P14 and then returned to room air. The mother rats were protected from the effects of hyperoxia exposure by exchanging them between the hyperoxia group and control group pups every 48 h. Each litter produced 10–12 pups, which were housed in cages with fostering mother rats. The experimental timeline is shown in Fig. 7.

### Behavioral assessment

Group allocation was blinded in all behavioral tests and evaluations. Negative geotaxis was conducted for 8 consecutive days, i.e., from P8 to P15, to assess the maturity of vestibular receptors, central sensory function, and motor function in neonatal rats^55^, and behavioral experiments were conducted during 5–7 weeks of age to assess general behavior, motor function, cognitive and memory abilities, and social skills in adult rats.

### Negative geotaxis

The rats were placed on an antislip mat on a 30° slope with their heads facing downward. The time taken by the rats to turn 180° and face upward was measured. Their performance was scored on a scale of 0–5 according to the time taken to make the 180° turn (score 5, 0–15 s; score 4, 15–30 s; score 3, 30–45 s; score 2, 45–60 s; score 1, > 60 s or drop; score 0, no response)^55^.

### Open field test

Open field test was conducted to assess general behavior, such as hyperactivity and behavioral abnormality^56^. In brief, rats were placed in the center of a 100 cm × 100 cm × 45 cm observation box and videographed for 5 min. The distance traveled and activity durations were measured using the ANY-maze Video Tracking System (version 4.99z, Stoelting Co., Wood Dale, IL, USA).

### Horizontal ladder walking test

Horizontal ladder walking test^57,58^ was conducted to assess motor deficits. Rats were made to walk on a ladder with randomly placed grids and videographed. We observed the frequency at which their hind legs failed to grasp the grid and calculated the ratio of missed steps out of all steps taken. This test was repeated three times, and the average ratio was reported. As pretraining before the final test, rats walked on ladders with evenly spaced grids three times a day for 3 consecutive days. Rats that were unable to complete the walk on the ladders were excluded from the analysis.

### Novel object recognition test

Novel object recognition test was performed to assess cognitive and memory abilities^59^. On the first day of the experimental session, rats were allowed to search for two identical objects in a 100 cm × 100 cm × 45 cm observation box for 5 min. On the second day of the experimental session, only one object was changed to a new object, and the rats were allowed to search for 5 min. To habituate the rats before the experimental session, they were allowed to explore the object-free apparatus for 5 min on 3 consecutive days. The exploratory behavior toward novel object was recorded using the ANY-maze Video Tracking System. The discrimination index was calculated from the measurements using Eq. (1):

1 $$Discrimination~Index=~\frac{{\left( {TN - TF} \right)}}{{\left( {TN+TF} \right)}}$$

where TF is the duration of familiar object exploration, and TN is the duration of novel object exploration.

To exclude freeze rats (a rat that curled up on the spot and stopped moving altogether), rats whose exploration duration for either object was < 3 s were excluded from the analysis.

### Social interaction test

Social interaction test^60^ was conducted to assess social skills. Pairs of unfamiliar and same group rats were simultaneously placed in a 100 cm × 100 cm × 45 cm observation box and videographed for 10 min. The duration of active interaction (social interaction time), i.e., sniffing, grooming, following, mounting, and climbing on/crawling under, was recorded.

### Histological and immunohistochemical analyses

To assess the damage induced by neonatal hyperoxia exposure, we evaluated changes occurring in the (1) acute phase (occurring within days of exposure) and (2) chronic phase (long-term effects, observed later in life).

The histological and immunohistochemical procedures were performed as previously described^61^ with minor modifications. In brief, in the acute phase, 5 mg/mL BrdU (Roche, Basel, Switzerland), dissolved in normal saline, was injected intraperitoneally once (at a dose of 50 mg/kg) at P6. Rat pups from both groups were randomly assigned to either P7 or P10, and these rats were administered a triad of anesthetics (medetomidine 0.2 mg/kg + midazolam 2 mg/kg + butorphanol 2.5 mg/kg) intraperitoneally under the influence of isoflurane inhalation anesthesia. The anesthetized rats were perfused intracardially, first with normal saline and next with 4% paraformaldehyde in phosphate-buffered saline (PBS). In the chronic phase, 8-week-old rats were anesthetized and perfused with 4% paraformaldehyde, as described above. Whole brains were collected from the paraformaldehyde-perfused rats. Subsequently, whole brains were immersion-fixed in 4% paraformaldehyde in PBS overnight at 4 °C. After immersion fixation, the cerebellum was separated from the whole brain and further into the right and left cerebellum at the midline of the cerebellum in sagittal sections. Subsequently, the right cerebellum was dehydrated with a graded series of ethanol and xylene, embedded in paraffin, and cut into 5-µm-thick sagittal sections, 30 and 50 μm apart, in acute and chronic phases, respectively. After deparaffinization and rehydration, antigen retrieval was performed by heating the sections for 10 min in 10-mM citrate buffer (pH 6.0). Nonspecific binding was blocked using 5% normal donkey serum in PBS with 0.3% Triton X-100. Subsequently, the sections were incubated overnight at 4 °C with monoclonal rat anti-BrdU (1:1,000 in PBS, Bio-Rad Laboratories, Hercules, CA), rat anti-MBP (1:200 in PBS, Merck Millipore, Burlington, MA), or rabbit anti-Calbindin (1:400 in PBS, Cell Signaling Technology, Danvers, MA) antibodies. The sections were subsequently incubated with appropriate biotinylated secondary antibodies (Vector Laboratories, Newark, CA) for 60 min at room temperature. Endogenous peroxidase activity was blocked by incubating the sections with 3% H_2_O_2_ (in PBS) for 10 min, and visualization was performed using an avidin–biotin–peroxidase solution (VECTASTAIN Elite ABC kit, Vector Laboratories). Peroxidase detection was then performed for 20 min (0.12 mg/mL 3,3-diaminobenzidine, 0.01% H_2_O_2_, and 0.04% NiCl_2_). To denaturalize DNA, sections for only BrdU staining were treated with 2 N HCl at 37 °C for 30 min, followed by incubation in 0.1-M borate buffer at room temperature for 10 min, before blocking with 5% normal donkey serum (in PBS with 0.3% Triton X-100).

For volume evaluation, paraffin sections were deparaffinized and stained with hematoxylin and eosin (HE) (Sakura Finetek Japan Co., Ltd., Chuo, Tokyo, Japan).

### Tissue evaluation and cell counting

#### Acute histological evaluation

Total volumes and total cell numbers were evaluated as previously described^62^. In brief, after outlining the borders of the external granular layer and cerebellar hemisphere in the HE-stained P7 cerebellar tissue sections using Stereo Investigator stereology software (version 2020, MicroBrightField, Williston, VT), the volume of the external granular layer and the total volume of the cerebellar hemisphere were determined according to the Cavalieri principle using Eq. (2):

2 $$V = \Sigma {\mathrm{A}} \times {\mathrm{P}} \times {\mathrm{T}}$$

where V is the total volume, ΣA is the sum of the measured areas, P is the inverse of the sampling fraction, and T is the section thickness. Subsequently, we calculated the ratio of the volume of the external granular layer to the total volume of the cerebellar hemisphere. The number of BrdU-positive cells in the inner margin of the external granular layer and those in the internal granular layer were counted using Stereo Investigator in BrdU-stained P10 cerebellar tissue sections. After outlining the borders of the inner margins of the external and internal granular layers, the computer program overlaid the outlined area with a grid system of counting frames. Cells within these frames and those touching two of the four predetermined sides of the frames were counted. The total number of BrdU-positive cells was calculated according to Cavalieri’s principle using Eq. (3):

3 $$N = \Sigma {\mathrm{n}} \times {\mathrm{P}}$$

where N is the total number of BrdU-positive cells, Σn is the sum of the number of positive cells in each section, and P is the inverse of the sampling fraction.

The volume of the counting area was determined using Eq. (2). Subsequently, we calculated the total number of BrdU-positive cells per mm^3^.

#### Chronic histological evaluation

Cerebellar volume was measured using Stereo Investigator in the HE-stained cerebellar tissue sections collected from 8-week-old rats, using Eq. (2). In addition, the proportion of the area positive for MBPs was measured using cellSens software (version 1.18, Olympus, Shinjuku, Tokyo, Japan) in immunohistochemically stained (with anti-MBPs antibodies) cerebellar tissue sections collected from 8-week-old rats. After outlining the region of interest, CellSens software was used to measure the proportion of areas expressing MBP. Furthermore, the number of Purkinje cells and length and diameter of Purkinje cell dendrites were measured in Calbindin-stained cerebellar tissue sections collected from 8-week-old rats. We measured the length to count five Purkinje cells, and the number of Purkinje cells per 100 μm was determined according to the following equation:

4 $$N=\frac{{500}}{A}$$

where N is the number of Purkinje cells per 100 μm and A is the length to count five Purkinje cells.

### Statistical evaluation

Statistical analyses were performed using Prism statistical software (version 10, GraphPad Software, Boston, MA). Independent sample *t*-tests were used to compare the two groups, with the assumption of population normality and variance uniformity. To analyze negative geotaxis scores over time between the two groups, repeated measures ANOVA was used, with time as a within-subject factor and group as a between-subject factor. An interaction term (Time × Group) was also included. When a significant interaction was found, post hoc pairwise comparisons were performed using independent sample *t*-tests and Bonferroni correction to adjust for multiple comparisons. A *p*-value of < 0.05 was considered statistically significant for repeated measures ANOVA, and an adjusted *p*-value of < 0.05 was used for post hoc tests. All results are presented as mean ± standard deviation and 95% CI.

## Supplementary Information

Below is the link to the electronic supplementary material.

Supplementary Material 1

Supplementary Material 2

Supplementary Material 3

## Data Availability

The datasets and materials generated during the current study are available from the corresponding author upon reasonable request.
