# Supplementary figures and images for: Neonatal hyperoxia exposure causes cerebellar lesions and behavioral abnormalities in rats
Source: Sci Rep. 2026 Jan 12;16:4289. doi: 10.1038/s41598-025-34530-1 (PMC12858802; doi:10.1038/s41598-025-34530-1)

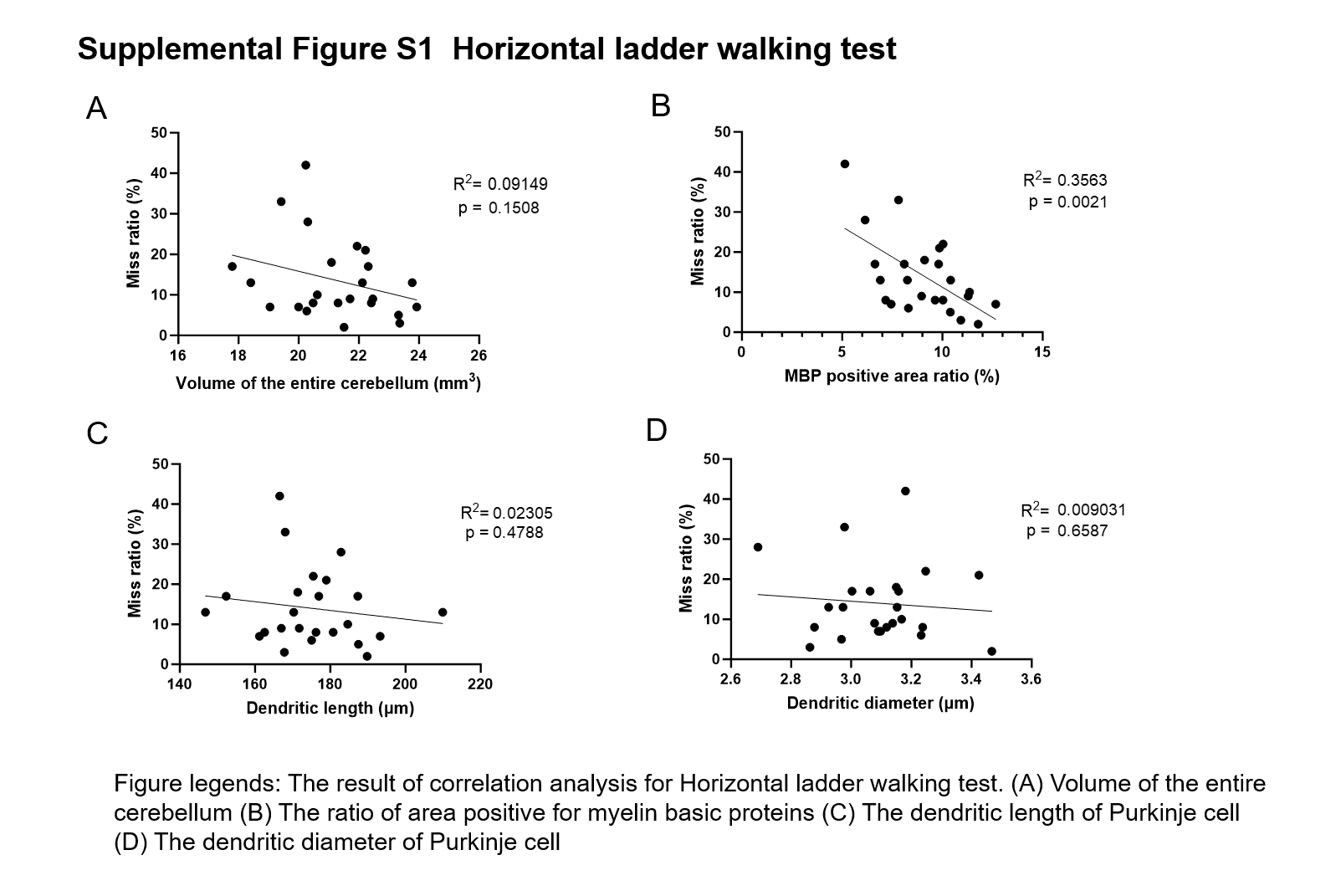

Supplement: Supplementary file 1 — Supplementary Material 1 [file 41598_2025_34530_MOESM1_ESM.tif]

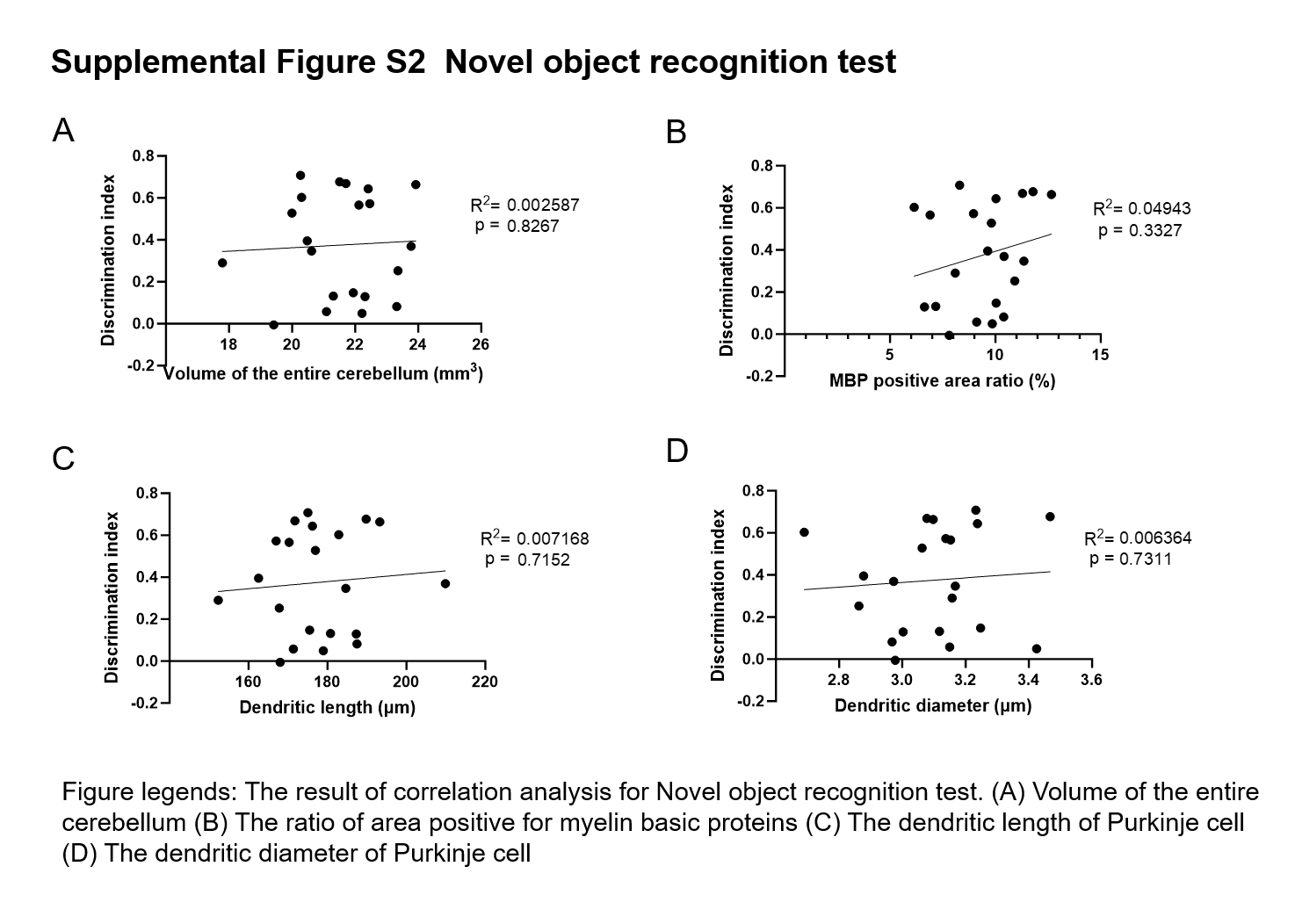

Supplement: Supplementary file 2 — Supplementary Material 2 [file 41598_2025_34530_MOESM2_ESM.tif]

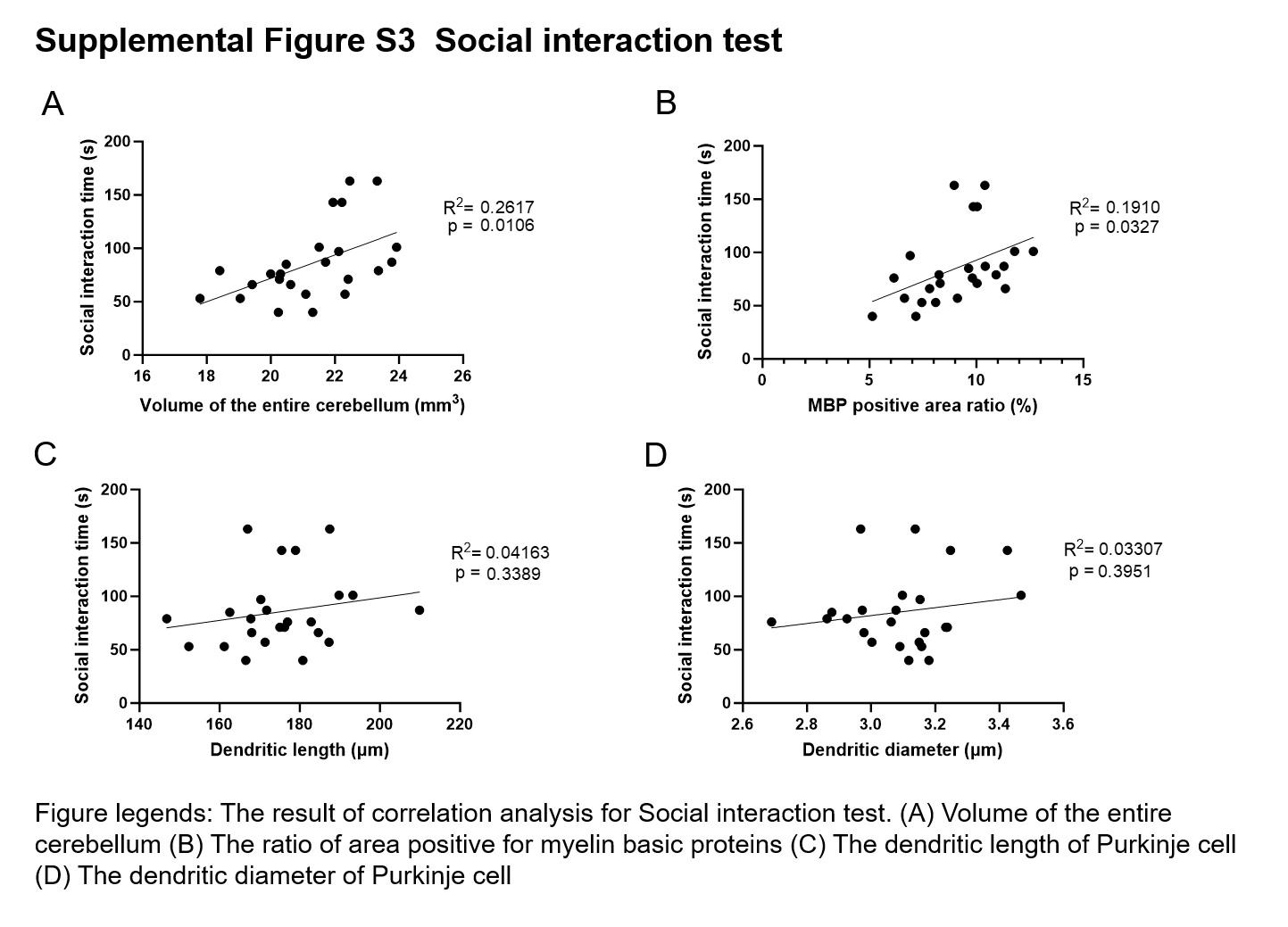

Supplement: Supplementary file 3 — Supplementary Material 3 [file 41598_2025_34530_MOESM3_ESM.tif]
